# The feasibility of the 1-h high-sensitivity cardiac troponin T algorithm to rule-in and rule-out acute myocardial infarction in Thai emergency patients: an observational study

**DOI:** 10.1186/s12245-018-0204-9

**Published:** 2018-10-22

**Authors:** Onlak Ruangsomboon, Pattaraporn Mekavuthikul, Tipa Chakorn, Apichaya Monsomboon, Nattakarn Prapruetkit, Usapan Surabenjawong, Chok Limsuwat, Sattha Riyapan, Wansiri Chaisirin

**Affiliations:** Department of Emergency Medicine, Siriraj Hospital Mahidol University, 2 Wangland Rd. Bangkoknoi, Bangkok, 10700 Thailand

**Keywords:** Acute myocardial infarction, High-sensitivity cardiac troponin T, 1-h hs-cTnT algorithm, Chest pain, Emergency department

## Abstract

**Background:**

The 3-h high-sensitivity cardiac troponin T (hs-cTnT) algorithm is the most commonly used scheme to diagnose acute myocardial infarction. The 1-h hs-cTnT algorithm has recently been approved by the European Society of Cardiology as an alternative algorithm for earlier diagnosis. If the hs-cTnT test cannot discriminate the diagnosis of the patient at 1 h, the patient is defined as observational group. Their test must be repeated at 3 h. A high prevalence of this group may indicate a low clinical utility of the 1-h hs-cTnT algorithm. This study was aimed to estimate the proportion of the observational group in Thai emergency department (ED) patients and also the time to rule-in/out between both the algorithms.

**Methods:**

A historical control study was conducted in patients with chest pain for 1–12 h at the ED of Siriraj Hospital, Bangkok, Thailand. The study compared two groups: one prospective group of all patients evaluated with the 1-h hs-cTnT algorithm between June and September 2017 and one historical control group evaluated with the 3-h hs-cTnT algorithm between January and March 2017.

**Results:**

A total of 130 patients were included (3-h hs-cTnT algorithm group *n* = 65 and 1-h hs-cTnT algorithm group *n* = 65). Twelve patients [18.5% (95% CI 10.0–30.1)] were categorized as observational group in the 1-h hs-cTnT algorithm group. The mean rule-in/out times in the 3-h hs-cTnT algorithm and 1-h hs-cTnT algorithm groups were 238 min (SD 63.3) and 134 min (SD 68.5), respectively (both *p* < 0.001). The time to disposition was also shortened in the 1-h hs-cTnT algorithm group (*p* <  0.001). Multivariable regression analysis performed to identify and adjust for confounders among patient characteristics revealed no significant confounders.

**Conclusions:**

The use of the 1-h hs-cTnT algorithm in the ED resulted in an acceptable proportion in the observational group and a decreased time to rule-in/out compared with the 3-h hs-cTnT algorithm.

## Background

Acute myocardial infarction (AMI) is one of the major causes of mortality [1, 2]. Cardiac biomarkers play an important role in the diagnostic process of non-ST-elevation myocardial infarction (NSTEMI) [3–6]. Of all cardiac biomarkers, cardiac troponin (cTn) is the most preferable due to its specificity to myocardial injury. It could provide high sensitivity and specificity for the diagnosis of NSTEMI [3, 4]. High-sensitivity cardiac troponin T (hs-cTnT) or high-sensitivity cardiac troponin I (hs-cTnI) could provide even higher sensitivity and specificity [7–11]. These biomarkers increased the diagnostic accuracy over conventional assay, especially in patients presenting early after chest pain onset [7–11]. In cases of NSTEMI, troponin will show a pattern of dynamic elevation above the 99th percentile [5]. Current clinical practice guidelines recommend a serial cardiac troponin test at presentation and 3 h later for evaluation of dynamic elevation compatible with myocardial injury [5, 6].

Earlier diagnosis and treatment of AMI can improve clinical outcome. Rapid exclusion is also helpful for the emergency department (ED) with limited resources or overcrowding. Recent approaches towards earlier diagnosis and exclusion using cutoff values below the 99th percentile have been proposed. The 1-h algorithm using hs-cTnT and hs-cTnI were developed and validated [12–17]. The 1-h hs-cTnT algorithm could substantially accelerate the management with accurate rule-in and safe rule-out for AMI in two large prospective cohort studies [16, 17]. In one multicenter study with more than 1200 patients enrolled with chest pain, the 1-h hs-cTnT algorithm could provide negative predictive value (NPV) and sensitivity for the rule-out group of as high as 99% and 97%, respectively [17]. Positive predictive value (PPV) and specificity for the rule-in group were also high at 77.2% and 96.1%, respectively [17]. The European Society of Cardiology (ESC) implemented the 1-h hs-cTnT or hs-cTnT algorithm in their 2015 guidelines for the management of non-ST-elevation acute coronary syndrome (NSTE-ACS), stating that the algorithms have been adequately validated and could be considered as an alternative in validated laboratory [6].

**Fig. 1:**
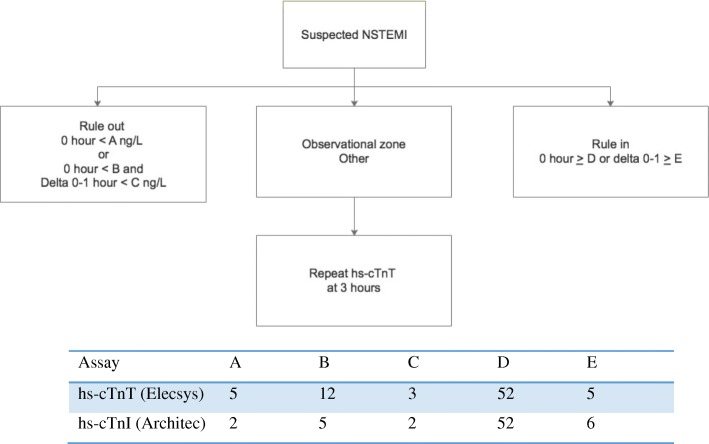
The hs-cTnT 1-h algorithm. Abbreviations: NSTEMI, non-ST-elevation myocardial infarction; hs-cTnT, high-sensitivity cardiac troponin T; hs-cTnI, high-sensitivity cardiac troponin I. Adapted from [6]

The 1-h hs-cTnT algorithm classifies patients into three groups: rule-in, rule-out, and observational group (Fig. 1). Patients who are triaged as the observational group are those who cannot be classified with hs-cTnT at 0 and 1 h. They require a third hs-cTnT test at 3 h, which is interpreted using the conventional 3-h hs-cTnT algorithm. Consequently, the observational group needs a total of three instead of two hs-cTnT tests. If the prevalence of this group is high, the putative improved cost-effectiveness of the 1-h hs-cTnT algorithm could be decreased. The prevalence of patients in the observational group in the previous prospective cohort studies ranged between 22 and 24% [12–17].

The 1-h hs-cTnT laboratory assay has only recently been certified for use at our institution. Although the 1-h hs-cTnT algorithm has been validated in many countries, its feasibility has not been studied in Thailand. We sought to estimate the prevalence of the observational group and the average change in time to rule-in/out after implementing the 1-h hs-cTnT algorithm.

## Methods

### Study design

The primary aim was to estimate the proportion of the observational group in the 1-h hs-cTnT algorithm. A high prevalence of the observational group may indicate that it has low feasibility in an often-crowded Thai ED setting. We recruited a 1-h hs-cTnT algorithm or post-implementation group prospectively for the primary aim.

The secondary aim was to compare the rule-in/out times between the 3 and 1-h hs-cTnT algorithms. We recruited a historical control or pre-implementation group that used the 3-h hs-cTnT algorithm retrospectively for the secondary aim. We assumed that ED crowding may be a confounding variable of time to rule-in/out. The variability of ED crowding in Siriraj Hospital usually depends on time of ED arrival. Therefore, the pre- and post-implementation groups were matched on time of the day and day of the week of presentation. Patients were divided into three groups based on their time of arrival: daytime of working day (8 am–8 pm, Monday to Friday), nighttime of working day (8 pm–8 am, Monday to Friday), and weekends or national public holidays.

### Setting

The study was conducted at the ED of Siriraj Hospital, Mahidol University, Bangkok, Thailand. The hospital is the largest tertiary and university hospital in Thailand with over 20,000 ED visits per year. The study was approved by the Siriraj Institutional Review Board (certificate of approval Si 328/2017). Informed consent was waived because the 1-h hs-cTnT algorithm was already validated, and the study did not affect rights, diagnostic adjustment, or clinical intervention of the patients.

### Participants

#### Eligibility criteria

Participants were included if they were over 18 years of age and presented to the ED with chest pain or other symptoms suggestive of AMI with the onset in a duration of 1–12 h prior to presentation. Participants were excluded if they had ST elevation on electrocardiogram (ECG), had undergone defibrillation or cardioversion in their visit to the ED, had undergone coronary artery bypass grafting within the last month, and had been diagnosed as AMI within the last 3 weeks. Patients were also excluded if they had stage V chronic kidney disease, had end-stage renal disease, were pregnant, or were breastfeeding.

#### Recruitment of the post-implementation group

All patients meeting the inclusion criteria were recruited prospectively and consecutively between 22 June and 12 September 2017. Investigations and treatments were given according to standard clinical practice guidelines. The 1-h hs-cTnT algorithm was conducted as shown in Fig. 1. After the patients were classified, they were managed according to their classification. Patients in the rule-out group were discharged if there were no other clinical problems. Patients in the rule-in group were treated as NSTEMI unless proven otherwise. The observational group had to wait for an additional hs-cTnT test at 3 h. Patients in this group were then classified as rule-in/out according to the 3-h hs-cTnT algorithm. Data were collected by medical record review after the patients were discharged.

#### Recruitment of the pre-implementation group

For this historical control group, medical records of patients presenting to the ED with chest pain or other symptoms suggestive of AMI with NSTE-ACS as the provisional diagnosis or a differential diagnosis from 8 January to 30 March 2017 were reviewed consecutively. We started with records on March 2017 and continued backwards in time until the quota of the comparable number of patients in each time of arrival interval was reached. In this group, the 3-h hs-cTnT algorithm was used to classify the patients. Investigations and management were also given according to standard clinical practice guidelines.

### Data collection

We collected the baseline patient demographics, the time of ED arrival, the time of first blood drawing, the troponin test results, the time of receiving the specimen at the laboratory, and the time of reporting results. The time of patient discharged from the ED, the patient disposition at discharge, and the medications for acute coronary syndrome (ACS) received by the patient were also collected. Major adverse cardiac events (MACE), defined as composite events of all-cause mortality, AMI, percutaneous or surgical revascularization, and significant stenosis on coronary angiography that occurred within 30 days of ED arrival, was also documented. Furthermore, the number of patients in the ED at the time that the patients arrived was recorded. This information was for the evaluation of the effect of ED crowding.

### Blood sample collection and laboratory diagnostic testing

At Siriraj Hospital, the blood specimens were sent to the central laboratory unit (ISO 15189 approved laboratory) using messengers. Samples in both groups were assayed using Elecsys 2010 solution in Cobas 8000 machine (Roche Diagnostics, Rotkreuz, Switzerland).

### Outcome measurements

#### Classification by the 1-h and 3-h hs-cTnT algorithms

According to either the 1- or 3-h hs-cTnT algorithm, the patients’ category (rule-in, rule-out, or observational group) after laboratory interpretation was recorded. In the observational group, the final category using the 3-h hs-cTnT result was recorded.

#### Proportion in the observational group using the 1-h hs-cTnT algorithm

This was the primary aim. The proportion was deemed acceptable if it was not more than that of the previous trials (22–24%) [12–17].

#### Average change in time to rule-in/out between the pre- and post-implementation groups

This was the secondary aim. We calculated time intervals. The rule-in/out time was defined as the time of first blood drawing to the time of the last laboratory result reporting. Laboratory transport time was defined as the time taken to bring the specimen to the laboratory. Laboratory processing time was defined as the time the laboratory took to analyze and interpret the results.

### Sample size calculation

For the primary aim, a previous pilot study in Siriraj Hospital investigated 10 patients using the 1-h hs-cTnT algorithm, and the proportion of the observational group was 0.4 (40%). With a proportion of 0.4 (95% CI 0.28–0.52), a sample of 65 was required. For the secondary aim, a pilot study investigated 10 patients using the 3-h hs-cTnT algorithm (group 1) and 10 patients using the 1-h hs-cTnT algorithm (group 2). The mean difference in time to rule-in/out was about 80 min with an SD in group 1 of 65 min and in group 2 of 30 min. A difference in mean time of 60 min was considered clinically important by the consensus of the authors. Using a two-sided type I error of 0.01 and 90% power, we calculated a sample of 25 per group. To answer primary and secondary aims, a sample size of 65 patients per group was required. nQuery Advisor (Cork, Ireland) was used to calculate the sample size.

### Statistical analysis

Data were reported using descriptive statistics and compared by chi-square and two-sample *t* tests as appropriate. Data with parametric distribution were reported as mean (SD). Data with nonparametric distribution were reported as median (IQR) and were compared by Mann-Whitney *U* test. Frequency was reported as number (%).

The proportion of observational group in the post-implementation group was reported with 95% CI. A two-sample *t* test was used to compare time to rule-in/out of AMI and other time intervals between the two groups. One-way ANOVA analysis was used to measure differences between subgroups. Multivariable linear regression was used to analyze factors influencing the secondary outcomes. Multivariable logistic regression was also used for triage type. Predictors were chosen by forward stepwise and backward stepwise selection. The variables examined included age, gender, onset of chest pain, current use of beta-blocker, nitrate, diuretic, ED prescription of aspirin, anticoagulant, nitroglycerin, intravenous furosemide, and oxygen treatment. All analyses used SPSS version 18 (SPSS Inc., Chicago, IL).

## Results

**Fig. 2:**
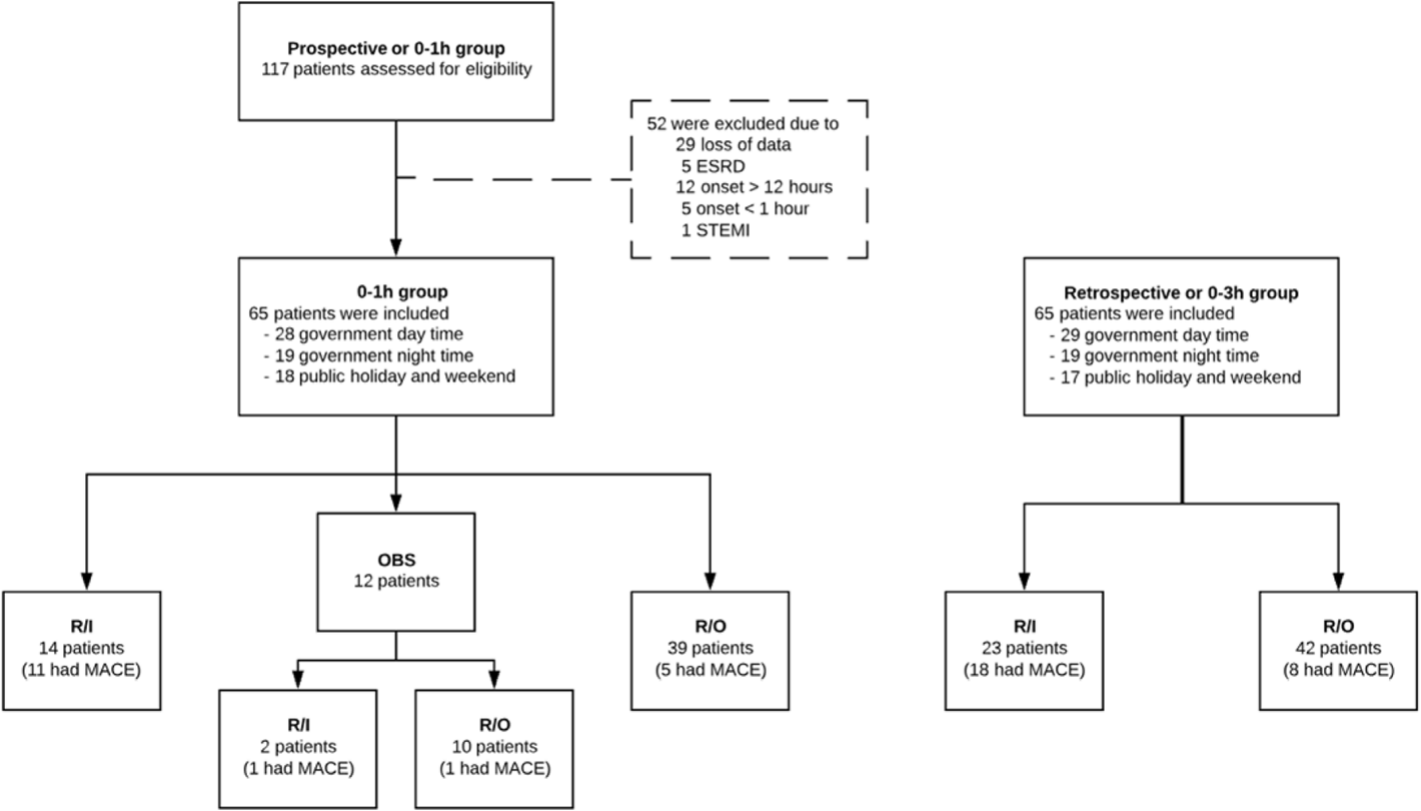
Flow diagram of study. Abbreviations: ESRD, end-stage renal disease; OBS, observational group; R/I, rule-in group; R/O, rule-out group, STEMI, ST-elevation myocardial infarction; 0–1 h, 1-h high-sensitivity cardiac troponin T algorithm; 0–3 h, 3-h high-sensitivity cardiac troponin T algorithm; MACE, major adverse cardiac events

A total of 130 patients were recruited. From June to September 2017, 65 patients were prospectively enrolled in the post-implementation group. In the pre-implementation group, 65 patients matched on the time of the day and day of the week during which they visited to the ED with the post-implementation group were enrolled during January and March 2017 (Fig. 2).

### Patient characteristics

Compared with the post-implementation group, the pre-implementation group was older and had longer onset of chest pain at presentation and lower glomerular filtration rate (GFR). They also had been taking more beta-blockers and diuretics prior to ED visit and had been given more treatments associated with ACS in the ED (Table 1).

**Table 1:** Patient characteristics

Variable	0–3 h (*n* = 65)	0–1 h (*n* = 65)	*p* value
Age (years)	71.6 ± 13.2	66.6 ± 14.2	0.041
Male sex	37 (56.9)	26 (40)	0.054
Underlying disease			
Coronary artery disease			
Previous CABG	12 (18.5)	10 (15.4)	0.77
Previous PCI	7 (10.8)	8 (12.3)	
No intervention	12 (18.5)	10 (15.4)	
Diabetes mellitus	25 (38.5)	21 (32.3)	0.46
Hypertension	45 (69.2)	43 (66.2)	0.71
Chronic kidney disease	27 (41.5)	23 (35.8)	0.47
Stage III	15 (23.1)	10 (15.4)	0.01
Stage IV	11 (16.9)	2 (3.1)	0.24
GFR (mL/min/1.73m^2^)	55.7 ± 25.8	67.1 ± 22.4	0.008
Current medication			
Aspirin	32 (49.2)	34 (5.2)	0.65
P2Y12 inhibitor	16 (24.6)	16 (24.6)	0.96
Beta-blocker	39 (58.5)	25 (38.5)	0.03
Calcium-channel blockers	23 (35.4)	20 (30.8)	0.62
Nitrate	24 (36.9)	13 (20)	0.05
Anticoagulant	9 (13.8)	10 (15.4)	0.78
Diuretic	17 (26.2)	8 (12.3)	0.007
ACEI/ARB	28 (43.1)	23 (35.4)	0.41
Onset of chest pain (hours)	5.5 ± 3.5	3.1 ± 3.0	< 0.001
Period of emergency department visit			
Government day time	28 (43.1)	29 (44.6)	0.99
Government night time	19 (29.2)	19 (29.2)	
Public holiday	18 (27.7)	17 (26.2)	
Initial electrocardiogram			
ST depression	15 (23.1)	11 (16.9)	0.54
T wave inversion	13 (20)	8 (12.3)	
Pathologic q wave	5 (7.7)	10 (15.4)	
LBBB	3 (4.6)	2 (3.1)	
RBBB	6 (9.2)	4 (6.2)	
Arrhythmia	5 (7.7)	7 (10.8)	
Treatment in emergency department			
Aspirin	27 (41.5)	15 (23.1)	0.04
P2Y12 inhibitor	24 (36.9)	17 (26.2)	0.19
Morphine	3 (4.6)	0 (0)	0.08
Nitroglycerine	23 (35.4)	14 (21.5)	0.08
Intravenous furosemide	28 (43.1)	18 (27.7)	0.07
Anticoagulant	26 (40)	19 (29.2)	0.2
Oxygen	37 (56.9)	17 (26.2)	< 0.001

Data was presented in mean ± SD. Frequency was presented in *n* (%)

*Abbreviations: ACEI* angiotensin converting enzyme inhibitor, *ARB* angiotensin receptor blocker, *CABG* coronary artery bypass grafting, *GFR* glomerular filtration rate, *LBBB* left bundle branch block, *PCI* percutaneous coronary intervention, *RBBB* right bundle branch block, *0–1 h* 1-h high-sensitivity cardiac troponin T algorithm, *0–3 h* 3-h high-sensitivity cardiac troponin T algorithm

### Primary outcome and secondary outcome

**Fig. 3:**
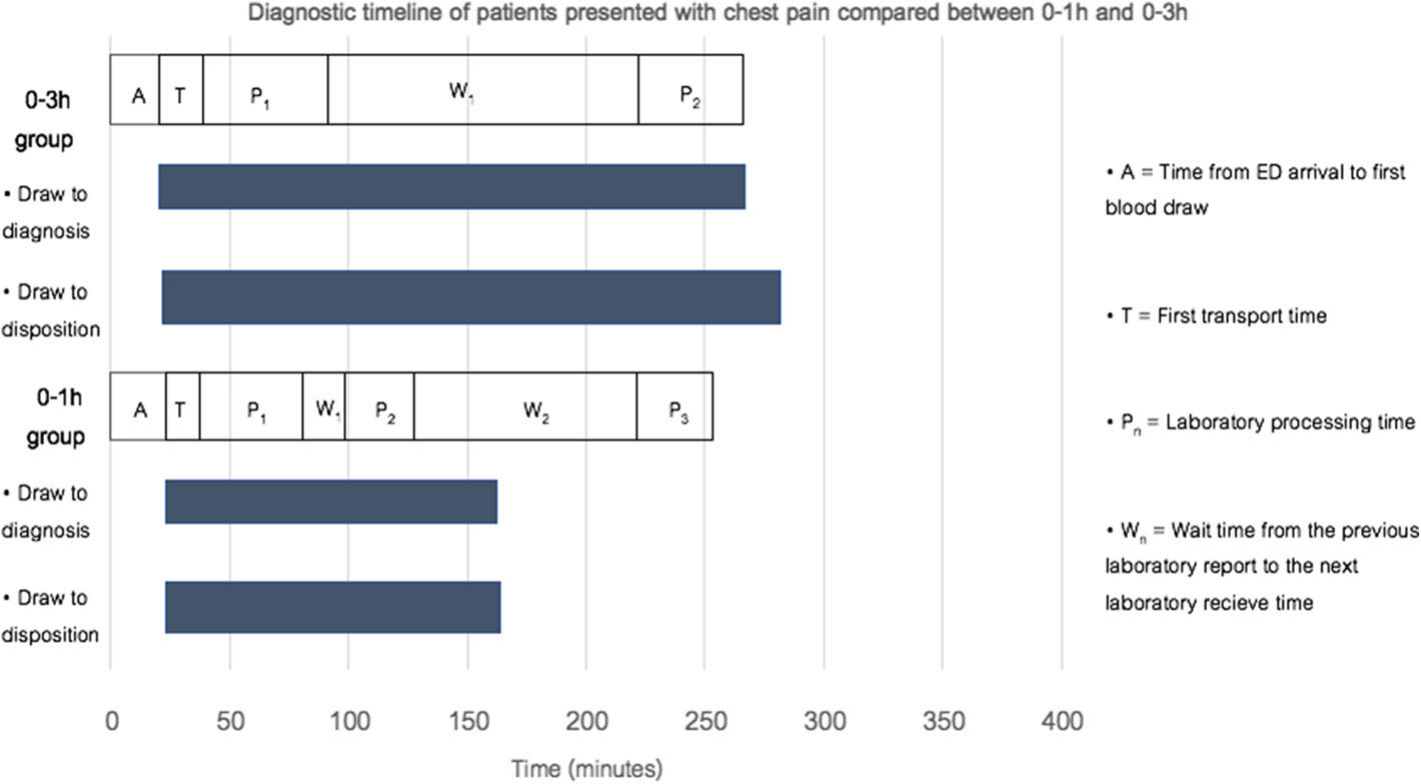
Diagnostic timeline of patients presenting to the ED with chest pain compared between 0–1 h and 0–3 h groups. Abbreviations: 0–1 h, 1-h high-sensitivity cardiac troponin T algorithm; 0–3 h, 3-h high-sensitivity cardiac troponin T algorithm

There were 12 patients [18.5% (95% CI 10.0–30.1)] categorized as the observational group in the post-implementation group. The mean rule-in/out time was 238 min (SD 63.3) in the pre-implementation group and 134.3 min (SD 68.5) in the post-implementation group (*p* < 0.001) (Table 2 and Fig. 3).

**Table 2:** Results

Variable	0–3 h (*n* = 65)	0–1 h (*n* = 65)	*p* value
Primary outcome			
Observational zone cohort	–	12 (18.5% (CI 10.0–30.1))	–
Secondary outcome			
Rule-in/out time (min)	238 ± 63.3	134.3 ± 68.5	< 0.001
Laboratory transport time (min)	18.3 ± 21.8	14.9 ± 13.6	0.29
Laboratory processing time (min)			
First laboratory	53.0 ± 16.6	43.1 ± 17.7	0.001
Secondary laboratory	44.5 ± 12.4	29.6 ± 10.9	< 0.001
Time to disposition (min)	260 (180–325)	140 (106.5–220)	< 0.001
Triage type and 30-day MACE			
Rule in	23 (35.4)	14 (21.5)	0.001
30-day MACE	18 (78.3)	11 (78.6)	
Sensitivity	69.2% (48.2, 85.7)	68.8% (41.1, 89)	
PPV	72% (55.6, 84.1)	73.3% (50.7, 88)	
Rule out	42 (64.6)	39 (60)	–
30-day MACE	8 (19)	5 (12.8)	
Specificity	82.1% (66.5, 92.5)	89.5% (75.2, 97.1)	
NPV	80% (68.8, 87.9)	87.2% (76.5–93.4)	
Observational zone	–	12 (18.5)	–
30-day MACE		2 (16.7)	
Disposition type			
Admit	20 (30.8)	11 (16.9)	0.065
Discharge	22 (33.8)	36 (55.4)	
Transfer to urgent room	20 (30.8)	17 (26.2)	
Refer	3 (4.6)	1 (1.5)	

Data was presented in mean ± SD, median (interquartile range) or 95% CI. Frequency was presented in *n* (%)

*Abbreviations: rule-in/out time* time to rule-in/out, *0–1 h* 1-h high-sensitivity cardiac troponin T algorithm, *0–3 h* 3-h high-sensitivity cardiac troponin T algorithm, *30-day MACE* major adverse cardiac events within 30 days, *PPV* positive predictive value, *NPV* negative predictive value

The mean laboratory transport time was not significantly different between the two groups. The mean laboratory processing time of the first specimen, that of the second specimen, and the median time to disposition were significantly longer in the pre-implementation group (*p* = 0.001, < 0.001, and < 0.001, respectively) (Table 2 and Fig. 3). After adjusting for baseline differences by multivariable linear regression, the adjusted differences of time to rule-in/out, laboratory transport time, and laboratory processing time were comparable to the crude mean differences. No patient characteristics had significant effect on any time interval from the regression model [min–max of *p* = 0.18–0.74].

The proportions of patients classified by the 3-h hs-cTnT algorithm in the pre-implementation group were rule-in in 35.4% and rule-out in 64.6%. The proportions of patients classified by the 1-h hs-cTnT algorithm in the post-implementation group were rule-in in 21.5% and rule-out in 60%. The number of patients in the observational group in the post-implementation group was 12 (18.5%). Of these, two patients were eventually ruled in according to the 3-h hs-cTnT algorithm. Both of them had further had cardiac testing. Of the 10 patients in the observational group who were eventually ruled out, two patients also had further cardiac testing. Of the observational group, two patients (16.7%) had 30-day MACE (Fig. 2). After categorizing the observational group into rule-in or rule-out, the triage type between the two groups was not significantly different (*p* = 0.25). Multivariable logistic regression model did not find any significant confounders among patient characteristics that predicted triage type. Disposition types were also not significantly different between the two groups (Table 2).

The mean number of patients in the ED was significantly higher on daytime of working day [11.0 (SD 3.2)] compared with nighttime of working day [8.7 (SD2.2)] and public holiday [8.6 (SD 2.9)] (*p* = 0.032 and 0.025, respectively). However, no significant differences in the mean rule-in/out time, laboratory processing time, laboratory transport time, and time to disposition by time of day and day of week were found in subgroup analysis (Table 3).

**Table 3:** Results compared by period of ED visit

Variable	0–3 h (*n* = 65)	0–1 h (*n* = 65)	*p* value
Rule-in/out time (min)			
Work D	260.5 ± 72.2 (*n* = 28)	131.5 ± 64 (*n* = 29)	< 0.001
Work N	226.4 ± 34.6 (*n* = 19)	146.8 ± 83.2 (*n* = 19)	< 0.001
Holiday	215.3 ± 63.6 (*n* = 18)	125.2 ± 61.4 (*n* = 17)	< 0.001
Laboratory processing time (min)			
Work D	58.5 ± 192 (*n* = 28)	45.9 ± 20.3 (*n* = 29)	0.19
Work N	51.0 ± 13.9 (*n* = 19)	41.8 ± 13.5 (*n* = 19)	0.05
Holiday	46.4 ± 12.2 (*n* = 18)	39.9 ± 17.2 (*n* = 17)	0.2
Laboratory transport time (min)			
Work D	26.0 ± 25.9 (*n* = 28)	15.6 ± 10.4 (*n* = 29)	0.05
Work N	12.2 ± 15.0 (*n* = 19)	13.5 ± 13.0 (*n* = 19)	0.77
Holiday	12.0 ± 17.7 (*n* = 18)	15.1 ± 18.9 (*n* = 17)	0.69
Time to disposition (min)			
Work D	280 (180, 327.5) (*n* = 28)	130 (109, 210) (*n* = 29)	< 0.001
Work N	225 (127.5, 307.5) (*n* = 19)	195 (96, 265) (*n* = 19)	0.59
Holiday	260 (187.5, 382) (*n* = 18)	135 (91.5, 192) (*n* = 17)	0.002

Data was presented in mean ± SD or median (interquartile range)

*Abbreviations: work D* daytime of working day, *work N* nighttime of working day, *holiday* public holiday and weekend, *rule-in/out time* time to rule in/out, *0–1 h* 1-h high-sensitivity cardiac troponin T algorithm, *0–3 h* 3-h high-sensitivity cardiac troponin T algorithm

## Discussion

The 1-h hs-cTnT algorithm is a validated alternative algorithm to the 3-h hs-cTnT algorithm for diagnosing AMI with the benefit of both earlier management for the rule-in group and earlier discharge for the rule-out group [4]. However, since the algorithm was developed based on earlier determination, it may lack the necessary properties to determine the observational group. To date, no studies have evaluated its use and the proportion of the observational group in Thailand. Therefore, we conducted this study to evaluate its feasibility.

The proportion of the observational group in our sample was 18.5%. The proportion was even less than in reports of previous trials, which were between 22 and 24% [12–17]. This was considered acceptable by our a priori definition of acceptable. Moreover, a reduction of approximately 100 min of ED length-of-stay when using the 1-h algorithm was useful. This proves the feasibility of this algorithm in crowded Thai ED settings. Nevertheless, further studies are necessary to estimate its cost savings.

We found that the number of patients visiting the ED was highest during daytime of working day. Although we hypothesized that ED crowding might cause a delay in the diagnostic process, the subgroup analysis with regard to period of ED visit showed no significant differences. A trend towards longer duration on daytime of working day was found in all intervals although these were not significant. This might have been because of small numbers of patients in subgroup analyses.

The laboratory processing time was longer in the pre-implementation group. This may have been caused by recent improvements in our laboratory analyzing system and process that affected the post-implementation group. However, in the post-implementation group, we found that the first laboratory process took about 13 min longer than the second. This might have been because the first laboratory examination usually consisted of not only cardiac troponin, but also other blood chemistry while the second laboratory taken 1 or 3 h later was usually troponin alone. Thus, the delay in the first laboratory process could have been from waiting for other blood chemistry results. If hs-cTnT had been analyzed and reported separately, the first laboratory processing time may have been similar to the second.

Patients in the pre-implementation group appeared to be more at risk of having an AMI. The differences may have been due to enrollment of controls who were matched using retrospective data collection. It might also have caused a time-related benefit for the pre-implementation group. Because of the prolonged time from the onset of chest pain to presentation, patients in this group had a higher tendency towards having highly elevated first hs-cTnT results, which would have made the diagnosis possible earlier than in the post-implementation group. Nevertheless, the outcomes after controlling for these patient characteristic differences were not significantly different from the original values. Disposition type was also not significantly different between the two groups despite the higher admission rate in the pre-implementation group. The reasons for this high admission rate were the higher number of rule-in patients predisposed to being admitted and the higher number of rule-out patients admitted due to diagnoses other than AMI in the pre-implementation group.

The 1-h hs-cTnT algorithm showed good discrimination to rule-in and rule-out patients suspected of AMI. The 1-h hs-cTnT algorithm had higher sensitivity, specificity, PPV, and NPV for 30-day MACE than the 3-h hs-cTnT algorithm. Nevertheless, this interpretation should be treated with caution given that each algorithm was performed on a different group of patients precluding direct comparison. Furthermore, the proportion of as much as 60% was seen in the rule-out category of the post-implementation group. The proportion that could be rapidly triaged as rule-in or rule-out at 1 h was as much as 81.5%. This percentage was comparable to the previous trials that externally validated this algorithm [12–17]. Nevertheless, the sensitivity, specificity, PPV, and NPV for 30-day MACE in our study were less than those for AMI in those previous trials [12–17]. This could be explained by the different outcome measure (MACE vs. AMI) and our small sample size that was inadequate to externally validate the algorithm’s ability to discriminate the classification of the patients because 30-day MACE was not planned as a study outcome.

### Limitations

There were many limitations in this study. First, the study design was a historical control design, which resulted in unequal patient characteristics. Although we controlled these in multivariable analysis, further studies properly matching by risk stratification should still be performed. Secondly, the sample size may have been too small to determine the effect of ED crowding. A further study with a large sample size may find a significant difference. Finally, this study was a single-centered study in a major university hospital tertiary care center. Thus, our findings may not be generalizable to other EDs in different Thai settings.

## Conclusions

The 1-h hs-cTnT algorithm was feasible because of the acceptable proportion in the observation group. It was beneficial for Thai ED patients with chest pain because it decreased the rule-in/out time compared with the 3-h hs-cTnT algorithm.

## Abbreviations

ACS: Acute coronary syndrome
AHA: American Heart Association
AMI: Acute myocardial infarction
cTn: Cardiac troponin
cTnT: Cardiac troponin T
ECG: Electrocardiogram
ED: Emergency department
ESC: European Society of Cardiology
GFR: Glomerular filtration rate
Holiday: Public holiday and weekend
hs-cTnI: High-sensitivity cardiac troponin I
hs-cTNT: High-sensitivity cardiac troponin T
MACE: Major adverse cardiac events
NPV: Negative predictive value
NSTE-ACS: Non-ST-elevation acute coronary syndrome
NSTEMI: Non-ST-elevation myocardial infarction
PPV: Positive predictive value
Work D: Daytime on working day
Work N: Nighttime on working day

## References

[1] World Health Organization (2014). Cardiovascular diseases: fact sheet number 310 [Internet].

[2] Centers of Disease Control and Prevention. Global health Thailand 2015 [Internet]. 2015. Available from: http://www.cdc.gov/globalhealth/countries/thailand/. [Cited 2017 Dec 27]

[3] Thygesen K, Mair J, Giannitsis E, Mueller C, Lindahl B, Blankenberg S (2012). How to use high-sensitivity cardiac troponins in acute cardiac care. Eur Heart J.

[4] Mueller C (2014). Biomarkers and acute coronary syndromes: an update. Eur Heart J.

[5] Amsterdam EA, Wenger NK, Brindis RG, Casey DE, Ganiats TG, Holmes DR (2014). 2014 AHA/ACC guideline for the management of patients with non ST-elevation acute coronary syndromes: executive summary: a report of the American College of Cardiology/American Heart Association Task Force on Practice Guidelines. Circulation.

[6] Roffi M, Patrono C, Collet J-P, Mueller C, Valgimigli M, Andreotti F (2016). 2015 ESC Guidelines for the management of acute coronary syndromes in patients presenting without persistent ST-segment elevation: Task Force for the Management of Acute Coronary Syndromes in Patients Presenting without Persistent ST-Segment Elevation of the European Society of Cardiology (ESC). Eur Heart J.

[7] Okamatsu K, Takano M, Sakai S, Ishibashi F, Uemura R, Takano T (2004). Elevated troponin T levels and lesion characteristics in non-ST-elevation acute coronary syndromes. Circulation.

[8] Reichlin T, Hochholzer W, Bassetti S, Steuer S, Stelzig C, Sc M (2009). Early diagnosis of myocardial infarction with sensitive cardiac troponin assays. N Engl J Med.

[9] Keller Till, Zeller Tanja, Peetz Dirk, Tzikas Stergios, Roth Alexander, Czyz Ewa, Bickel Christoph, Baldus Stephan, Warnholtz Ascan, Fröhlich Meike, Sinning Christoph R., Eleftheriadis Medea S., Wild Philipp S., Schnabel Renate B., Lubos Edith, Jachmann Nicole, Genth-Zotz Sabine, Post Felix, Nicaud Viviane, Tiret Laurence, Lackner Karl J., Münzel Thomas F., Blankenberg Stefan (2009). Sensitive Troponin I Assay in Early Diagnosis of Acute Myocardial Infarction. New England Journal of Medicine.

[10] Giannitsis E, Becker M, Kurz K, Hess G, Zdunek D, Katus HA (2010). High-sensitivity cardiac troponin T for early prediction of evolving non-ST-segment elevation myocardial infarction in patients with suspected acute coronary syndrome and negative troponin results on admission. Clin Chem.

[11] Cullen L, Aldous S, Than M, Greenslade JH, Tate JR, George PM (2014). Comparison of high sensitivity troponin T and I assays in the diagnosis of non-ST elevation acute myocardial infarction in emergency patients with chest pain. Clin Biochem.

[12] Rubini GM, Twerenbold R, Jaeger C, Schindler C, Puelacher C, Wildi K (2015). One-hour rule-in and rule-out of acute myocardial infarction using high-sensitivity cardiac troponin I. Am J Med.

[13] Druey S, Wildi K, Twerenbold R, Jaeger C, Reichlin T, Haaf P (2015). Early rule-out and rule-in of myocardial infarction using sensitive cardiac Troponin I. Int J Cardiol.

[14] Neumann JT, Sörensen NA, Ojeda F, Renné T, Schnabel RB, Zeller T (2017). Early diagnosis of acute myocardial infarction using high-sensitivity troponin I. PLoS One.

[15] Reichlin Tobias, Schindler Christian, Drexler Beatrice, Twerenbold Raphael, Reiter Miriam, Zellweger Christa, Moehring Berit, Ziller Ronny, Hoeller Rebeca, Rubini Gimenez Maria, Haaf Philip, Potocki Mihael, Wildi Karin, Balmelli Cathrin, Freese Michael, Stelzig Claudia, Freidank Heike, Osswald Stefan, Mueller Christian (2012). One-Hour Rule-out and Rule-in of Acute Myocardial Infarction Using High-Sensitivity Cardiac Troponin T. Archives of Internal Medicine.

[16] Reichlin T, Twerenbold R, Wildi K, Rubini Gimenez M, Bergsma N, Haaf P (2015). Prospective validation of a 1-hour algorithm to rule-out and rule-in acute myocardial infarction using a high-sensitivity cardiac troponin T assay. CMAJ.

[17] Mueller C, Giannitsis E, Christ M, Ordóñez-Llanos J, de Filippi C, McCord J (2016). Multicenter evaluation of a 0-hour/1-hour algorithm in the diagnosis of myocardial infarction with high-sensitivity cardiac troponin T. Ann Emerg Med.

